# Case Report: Allograft aortic valve replacement in irreparable infant mitral valve

**DOI:** 10.3389/fcvm.2024.1425900

**Published:** 2024-07-24

**Authors:** Yuqing Niu, Shaoxian Cheng, Nianguo Dong, Cheng Zhou

**Affiliations:** ^1^ Department of Cardiovascular Surgery, Union Hospital, Tongji Medical College, Huazhong University of Science and Technology, Wuhan, China; ^2^ Jingshan Union Hospital, Union Hospital, Tongji Medical College, Huazhong University of Science and Technology, Wuhan, China

**Keywords:** mitral valve repair, mitral regurgitation (MR), mitral valve disease, allograft aortic valve, infants

## Abstract

This case report describes a 3-month-old male infant diagnosed with severe mitral stenosis (MS) and mitral regurgitation (MR) by transthoracic echocardiography. The male infant initially underwent complex mitral valve repair surgery. However, postoperative deterioration occurred with hemodynamic instability and shock, necessitating multiple resuscitation efforts and ultimately requiring support from Extracorporeal Membrane Oxygenation (ECMO). Given the serious conditions, the cardiac team decided to perform mitral valve replacement with a fresh allograft aortic valve. Postoperatively, the patient was promptly weaned off ECMO support, and the valve demonstrated sustained functionality throughout the long-term follow-up.

## Introduction

Mitral valve repair is the preferred treatment for infants with severe mitral regurgitation MR (1). If mitral valve repair yields an unfavorable prognosis, characterized by persistent severe regurgitation or other complications, additional surgeries such as re-repair or valve replacement may become necessary (2). However, traditional mitral valve replacement surgery poses multiple challenges for infants, including a high mortality rate, size mismatch of the mechanical valve with the growing heart, and lifelong anticoagulant therapy, complicating its application in this population (3–5). Consequently, there is a need for advanced valve replacement techniques for infants. This report details the case of an infant who initially underwent mitral valve repair for regurgitation, and subsequently underwent mitral valve replacement using an allograft aortic valve due to worsening symptoms.

## Case presentation

**Figure 1:**
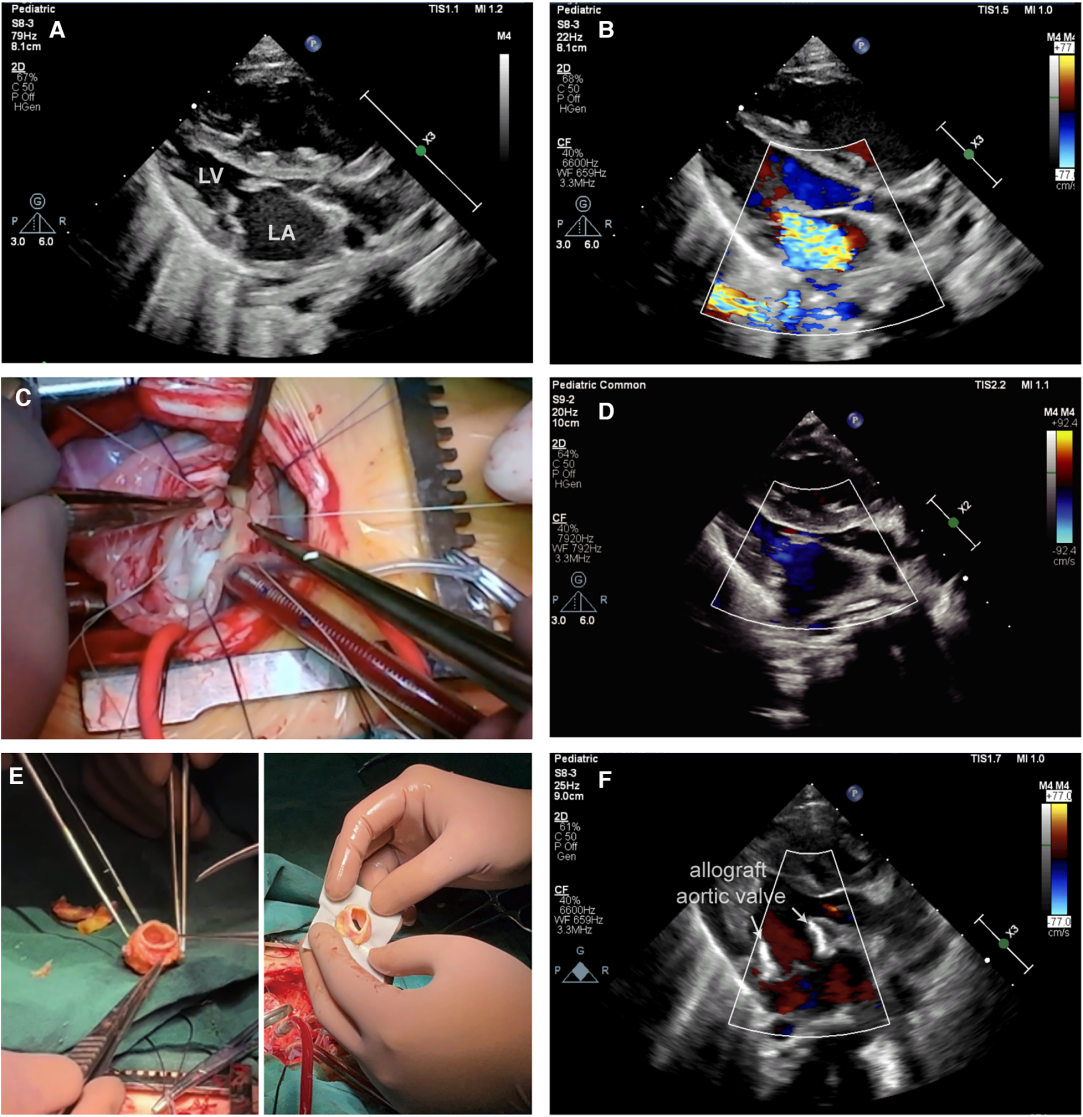
(**A**) Transthoracic echocardiography (TTE) demonstrating mitral stenosis in conjunction with severe closure insufficiency. (**B**) TTE demonstrated severe mitral regurgitation before surgery. (**C**) This image shows a complex mitral valve repair in operation. (**D**) Postoperative TTE showed moderate mitral regurgitation. (**E**) Left: This figure shows a trimmed fresh allograft aortic valve. Right: This figure illustrates the implantation of a fresh allograft aortic valve in the mitral position using the Top-hat method. (**F**) Postoperative TTE shows a well-suited and well-functioning mitral valve with mild mitral regurgitation following allograft aortic valve (arrow) replacement.

A 3-month-old male infant, 5.35 kg, was diagnosed with severe MS and MR via transthoracic echocardiography (Figures 1A,B). Preoperative echocardiography revealed right heart and left atrial enlargement, with the left ventricle compressed into a “D” shape. The mitral valve is significantly thickened, with hypertrophic and underdeveloped papillary muscles and thickened, shortened chordae tendineae. The valve leaflets are stiff, with restricted opening (orifice area −1.0 cm^2^) and poor closure. The tricuspid valve is also thickened, with thickened and shortened sub-valvular chordae tendineae and poor closure. Color Doppler flow imaging (CDFI) revealed accelerated diastolic blood flow through the mitral valve with a peak velocity of 2.7 m/s, a pressure gradient of 29 mmHg, and a mean pressure gradient of 13 mmHg. Significant regurgitation signals are observed on the left atrial side during systole for the mitral valve and on the right atrial side during systole for the tricuspid valve. To address these conditions, the patient underwent a complex mitral valvuloplasty procedure under anesthesia (Figure 1C). The mitral valve repair procedure includes several key steps: firstly, the abnormal chordae tendineae are excised, followed by commissurotomy and papillary muscle release. Subsequently, artificial chordae tendineae are placed, and repair is performed using the commissure magic stitch technique (Supplementary Video S1).

Unfortunately, on the second day after surgery, the condition of the infants deteriorated to severe cardiogenic shock, necessitating the use of extracorporeal membrane oxygenation (ECMO) for life support. Subsequent postoperative echocardiography revealed worsening MR with extensive regurgitation (Figure 1D).

In response to this critical situation, a second surgical intervention, mitral valve replacement (MVR), was planned. A freshly harvested aortic valve from another pediatric heart transplant recipient was preserved at 4°C in an antibiotic-containing culture medium for 10 days and used for this procedure (Figure 1E, left). This innovative approach was approved by the Ethics Committee of Wuhan Union Hospital. The allogeneic aortic valve was anastomosed to the mitral annulus utilizing the Top-Hat technique (Figure 1E, right). In this procedure, we first removed the artificial chordae tendineae and leaflet patches of the mitral valve, which were sized to pass through a size 17 dilator. We then thoroughly rinsed and trimmed the allograft aortic valve, suturing the left and right coronary artery openings. The allograft aortic valve was measured to be passable by a size 17 dilator. Next, using 6-0 Prolene sutures, we performed continuous stitching to precisely attach the bovine pericardial cuff to the base of the aortic valve. Following this, we used 6-0 Prolene sutures in a continuous and interrupted pattern to accurately secure the distal end of the aortic valve to the mitral valve annulus. Finally, to maintain normal pulmonary vein return, we employed 6-0 Prolene suture in a continuous and interrupted manner to precisely attach the bovine pericardial cuff to the left atrial wall (Supplementary Video S2). Remarkably, the patient was successfully weaned off ECMO support immediately after surgery, and postoperative echocardiography confirmed a reduction in MR to a trace amount (Figure 1F). Postoperative follow-up echocardiography revealed a strong echogenic signal from the allograft valve framework at the mitral position, with normal leaflet mobility. Color Doppler flow imaging (CDFI) showed no significant diastolic flow acceleration through the allograft valve. Blood flow through the mitral valve exhibited a peak velocity of 1.9 m/s, with a pressure gradient of 14 mmHg and a mean pressure gradient of 6 mmHg. Mild to moderate regurgitation was observed during systole, with no abnormal paravalvular regurgitation detected. After this surgery, the mean pressure gradient across the mitral valve decreased from 14 mmHg to 6 mmHg, indicating successful improvement of the patient's cardiovascular condition. Throughout the seven-month follow-up period, the infant exhibited favorable growth, and the transplanted valve continued to function effectively.

## Discussion

Congenital mitral valve abnormalities in infants are rare cardiac anomalies, with an incidence of approximately 5 per 100,000 live births, frequently presenting alongside other intracardiac malformations (6, 7). The etiology of mitral valve disease (MVD) differs in adults and children. In adults, common causes include infective endocarditis, rheumatic diseases, valve calcification, connective tissue diseases such as systemic lupus erythematosus and scleroderma, and ischaemic cardiomyopathy (8–10). In children, mitral valve disease is mainly caused by congenital factors and infectious diseases (6, 11). According to intraoperative pathological findings, the infant in this case was mainly considered to have a congenital developmental abnormality of the mitral valve. Furthermore, this infant presented with severe congenital mitral stenosis accompanied by regurgitation, and the lesions involved the leaflets and sub-valvular structures making it the most difficult category of mitral valve repair. Infants with MS commonly exhibit pulmonary hypertension and right ventricular failure, potentially leading to developmental delays (12). Additionally, mitral regurgitation in infants can lead to pneumonia, feeding difficulties, and stunted growth, with the risk of respiratory and cardiac failure as the condition deteriorates (13).Consequently, the emergence of symptoms typically necessitates prompt intervention.

Congenital mitral valve disease poses challenges in terms of treatment, particularly in surgical treatment. The complexity of such operations is attributed to factors such as restricted operating space and the fragility of cardiac tissues. Current guidelines favor mitral valve repair over replacement in managing mitral valve disease (1). Research indicates that approximately 90% of pediatric mitral valvuloplasty patients experience positive short-term outcomes (14). However, a portion of pediatric patients may persistently experience moderate to severe MR after surgery, which is attributed to less-than-ideal repair results, natural disease progression, prosthetic material complications, and valve dysfunction recurrence (15). Consequently, additional interventions, including re-repair or valve replacement, become essential to enhance patient outcomes.

Traditional MVR in infants presents unique challenges, including stenosis due to the limited growth capacity of prosthetic valves and the requirement for long-term anticoagulation with mechanical valves (16). Additionally, the risk of complications and mortality is elevated in infants following MVR. A stent-mounted, valved, bovine jugular vein graft (Melody valve), approved for transcatheter pulmonary valve replacement, was adapted for mitral implantation in 2010. Although it demonstrated acceptable short-term function and could be expanded via catheter-based procedures as the child grows, we did not consider this approach due to the risks of mortality and structural valve deterioration. Furthermore, this technology is not widely used in our country, and the Melody valve is not available in the Chinese market. Therefore, exploring innovative MVR methods is crucial when repair is untenable.

To the best of our knowledge, this is the first case in the literature to demonstrate the feasibility of MVR with an allograft aortic valve in instances where the mitral valve is irreparable in infants. Postoperatively, the patient was promptly weaned off ECMO support, and the valve demonstrated sustained functionality throughout the long-term follow-up. This procedure addresses significant challenges due to the limited availability of appropriately sized prosthetic valves for the small mitral annulus in pediatric patients and the inherent lack of growth potential in existing cardiovascular prosthetic materials. It is undeniable that such procedures cannot be performed as standard, yet this technique can prove highly effective in specific circumstances, meriting careful consideration.

## Conclusion

This case report demonstrates the feasibility and effectiveness of performing MVR with an allograft aortic valve in instances where the mitral valve is irreparable in infants.

## Data Availability

The original contributions presented in the study are included in the article/Supplementary Material, further inquiries can be directed to the corresponding authors.
